# The Role of the Gut Microbiome in Pediatric Obesity and Bariatric Surgery

**DOI:** 10.3390/ijms232315421

**Published:** 2022-12-06

**Authors:** Cynthia Omoge Akagbosu, Evan Paul Nadler, Shira Levy, Suchitra Kaveri Hourigan

**Affiliations:** 1 Division of Pediatric Gastroenterology, Children’s National Hospital, 111 Michigan Ave. NW, Washington, DC 20010, USA; 2 Division of Pediatric Surgery, Children’s National Hospital, 111 Michigan Ave. NW, Washington, DC 20010, USA; 3 Clinical Microbiome Unit (CMU), Laboratory of Host Immunity and Microbiome, National Institute of Allergy and Infectious Diseases, National Institutes of Health, Bethesda, MD 20892, USA

**Keywords:** gut microbiota, gut microbiome, pediatric obesity, bariatric surgery, sleeve gastrectomy, obesity treatment, microbiota manipulation, dysbiosis, weight management

## Abstract

Obesity affects 42.4% of adults and 19.3% of children in the United States. Childhood obesity drives many comorbidities including hypertension, fatty liver disease, and type 2 diabetes mellitus. Prior research suggests that aberrant compositional development of the gut microbiome, with low-grade inflammation, precedes being overweight. Therefore, childhood may provide opportunities for interventions that shape the microbiome to mitigate obesity-related diseases. Children with obesity have gut microbiota compositional and functional differences, including increased proinflammatory bacterial taxa, compared to lean controls. Restoration of the gut microbiota to a healthy state may ameliorate conditions associated with obesity and help maintain a healthy weight. Pediatric bariatric (weight-loss) surgery is an effective treatment for childhood obesity; however, there is limited research into the role of the gut microbiome after weight-loss surgery in children. This review will discuss the magnitude of childhood obesity, the importance of the developing microbiome in establishing metabolic pathways, interventions such as bariatric surgery that may modulate the gut microbiome, and future directions for the potential development of microbiome-based therapeutics to treat obesity.

## 1. Introduction

Childhood obesity is an epidemic in the United States; 19.3% of children have obesity and 6.1% have severe obesity [1]. Severe childhood obesity is associated with complications including hypertension, fatty liver disease, and type 2 diabetes mellitus [2,3]. If individuals can achieve weight loss prior to entering adulthood, the risk of these conditions is mitigated [4], making childhood obesity a key target area for intervention. Bariatric (weight-loss) surgery is highly effective in enabling weight loss and decreasing associated comorbidities, although the biological mechanisms underlying weight loss in bariatric surgery are not fully elucidated. The gut microbiota (community of microbes in the intestines) appears to provide bacterial control of metabolic processes that could impact energy regulation in obesity, but its role has not yet been determined following bariatric surgery in children. It is important to study the gut microbiota in children as the developing microbiome of a child differs from the microbiome of an adult and plays a clearer role in establishing metabolic pathways. This review aims to characterize the gut microbiome in healthy children, the role of gut microbiome dysbiosis in obesity, and to discuss strategies to modify disease-prone gut microbiota in children with severe obesity, including bariatric surgery.

## 2. Epidemiological Factors in Obesity

### 2.1. Definition of Obesity in Children and Adults

Body mass index (BMI) is used as a screening tool for both childhood and adult obesity and is defined as weight in kilograms divided by the square of height in meters [2]. A BMI of 18.5 kg/m^2^ to 24.9 kg/m^2^ is considered a normal or healthy weight [1]. In adults, overweight is defined as BMI ≥ 25 kg/m^2^ with class 1 obesity defined as BMI ≥ 30 kg/m^2^, class 2 obesity BMI ≥ 35 kg/m^2^, and class 3 obesity BMI ≥ 40 kg/m^2^. Class 3 obesity is also termed severe obesity [2]. In contrast, a child’s body composition changes during growth from infancy into adulthood, with children growing at different rates at different times [1]. Therefore, for ages 2 through 19, weight status is calculated in comparison with other same-age and same-sex children using specific growth charts compiled by the Center For Disease Control and Prevention (CDC) [1]. For children less than 2 years of age, weight-for-length should be plotted using the World Health Organization (WHO) normative growth charts [5]. A normal or healthy weight is defined as the 5th percentile to less than the 85th percentile [1], with overweight defined as BMI ≥ 85th percentile, and class 1 obesity defined as BMI ≥ 95th percentile [2]. The adaptation of adult criteria for obesity unto children includes class 2 obesity (≥120% of the 95th percentile) and class 3 obesity (≥140% of the 95th percentile), with severe obesity generally describing a child’s BMI ≥ 120% of the 95th percentile [2].

### 2.2. Prevalence of Obesity

The National Health and Nutrition Examination Survey (NHANES) from 2017–2018 estimates that in adults, 30.7% are overweight, 42.4% have obesity, and 9.2% have severe obesity [1]. From the same survey, 16.1% of children and adolescents are overweight, 19.3% have obesity, and 6.1% have severe obesity [1]. Based on these numbers, the prevalence of childhood obesity doubled over the past 30 years [1,6]. Furthermore, weight gain among children and adolescents increased during the COVID-19 pandemic [7], highlighting a need for effective interventions that can be delivered remotely [8].

### 2.3. Differences in Obesity Rates Based on Race/Ethnicity and Age/Sex

**Figure 1:**
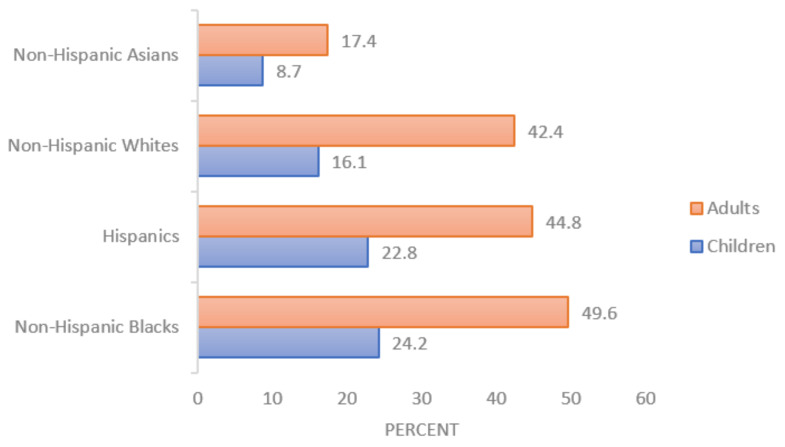
Prevalence of obesity among children and adults, by race and ethnicity in the United States; adapted from 2017–2018 NHANES data [1,9].

There are disparities among racial/ethnic groups in terms of obesity prevalence. Non-Hispanic Black women are the only adult population (by race/ethnicity and sex) with over half of the constituents having obesity, at 56.9% [1]. Obesity in adults is highest among non-Hispanic Blacks (49.6%), followed by Hispanics (44.8%), non-Hispanic Whites (42.4%), and non-Hispanic Asians (17.4%). Severe obesity in adults is highest among non-Hispanic Blacks (13.8%), followed by non-Hispanic Whites (9.3%), Hispanics (7.9%), and non-Hispanic Asians (2.0%) [1]. These disparities in obesity rates are also present in, and likely stem from, the pediatric population (Figure 1). Non-Hispanic Black girls are again the population (by race/ethnicity and sex) with the highest amount of obesity, at 29.1% [1]. Obesity in children is highest among non-Hispanic Blacks (24.2%), followed by Hispanics (22.8%), non-Hispanic Whites (16.1%), and non-Hispanic Asians (8.7%) [9]. Severe obesity in children is highest among non-Hispanic Blacks (10.2%), followed by Hispanics (5.3%), non-Hispanic Whites (4.3%), and non-Hispanic Asians (2.1%) [9].

## 3. Obesity-Related Comorbidities in Children and Adults

Childhood obesity is linked to hypertension, fatty liver disease, type 2 diabetes mellitus, sleep apnea, orthopedic disorders, and poor psychological health [2,3]. These same comorbidities are found in adults who have obesity [3]. The etiology of childhood obesity is multifactorial, including environmental, behavioral, genetic, dietary, microbial, and biological contributions [2,3].

Juonala et al., examined 6328 subjects from four prospective cohort studies (2 in the United States, 1 in Australia, 1 in Finland) that measured childhood and adult BMI for an average follow-up time of 23 years [4]. They found that adults with normal weight who suffered from overweight or obesity during childhood, had similar risks of adverse metabolic health outcomes to adults who had a normal weight during childhood. The authors also found that of the children with normal weight, only 14.6% developed obesity as adults, of the children with an overweight status, 64.6% developed obesity as adults, and of the children who had obesity, 82.3% continued to have obesity in adulthood [4], which was also seen in other population-based data [10].

Children with obesity who later entered adulthood with obesity faced a significantly increased risk of cardiometabolic events [4]. However, if a child with obesity entered adulthood at a normal weight, their risk returned to that of any other adult who possessed a normal weight their entire life [4]. Thus, early intervention is vital to curtailing the obesity epidemic. Of note, this study did not detail which, if any, specific interventions helped children with obesity develop a normal weight as an adult.

## 4. Bariatric Surgery as a Treatment for Childhood Obesity

Obesity treatments include lifestyle interventions, medications, and bariatric surgery. Current pediatric obesity treatment algorithms no longer focus on lifestyle modifications alone [3,11,12]. The following medications, listed by date of approval, provide some effectiveness in adult populations, with expanding use in children [13]. Orlistat, one of the earlier Food and Drug Administration (FDA) approved medications for weight loss in individuals aged 12 and older, is rarely used in current clinical practice due to unpleasant side effects such as flatulence [3]. Metformin is a drug used in diabetes management that also promotes weight loss in children who have insulin resistance [11]. However, it is not approved by the FDA for the sole indication of weight reduction, and generally does not result in significant weight loss when taken alone [11]. Liraglutide is a glucagon-like peptide 1 (GLP-1) agonist whose approval was extended to adolescents aged 12 to 17 in 2020 [14]. While its use in the pediatric population is increasing, many patients decline initiation of the drug since it requires a daily subcutaneous injection. Setmelanotide, approved in 2021, is one of the first FDA-approved medications for chronic weight management in genetic causes of obesity including pro-opiomelanocortin (POMC), proprotein convertase subtilisin/kexin type 1 (PCSK1), and leptin receptor (LEPR) deficiencies [15]. However, these monogenic causes of obesity are rare and the utility of this medication to treat other types of obesity remains unknown. More recently, the combination of phentermine/topiramate (Qsymia) has been approved in pediatric patients aged 12 years and older as of 2022 but has side effects like dizziness [16]. One promising drug, currently approved for adults, is semaglutide, a GLP-1 agonist that is only administered once-weekly (rather than daily as with liraglutide) [17]. In November 2022, Weghuber et al. published a clinical trial where semaglutide garnered significant weight reduction in adolescents (pending FDA approval for this age group) [17]. In an adult head-to-head comparison trial, semaglutide was significantly more effective than liraglutide in terms of mean weight reduction (15.8% vs. 6.4%, respectively) [18]. All the medications listed above have risks and benefits, but the overall weight loss associated with their use is far less than the weight loss seen after bariatric surgery, which has led to increased utilization of bariatric surgery in the pediatric age groups [3,12].

According to the American Society for Metabolic and Bariatric Surgery (ASMBS) Pediatric Metabolic and Bariatric Surgery Guidelines from 2018, bariatric surgery is a potential intervention for children and adults with class 2 obesity and comorbidities or class 3 obesity with or without comorbidities [19]. This guideline and most others derive from the 1991 NIH Consensus Statement for Gastrointestinal Surgery in Severe Obesity [20,21]. However, the 2022 ASMBS and International Federation for the Surgery of Obesity and Metabolic Disorders (IFSO) now recommend lower BMI thresholds for adult bariatric surgery: class 1 obesity with comorbidities that do not improve using nonsurgical methods and class 2 obesity with or without comorbidities [21].

Bariatric surgery is considered the gold standard treatment for severe obesity and is the most effective option in both adolescents and adults, but there are some concerns regarding long-term efficacy [22,23]. The American Academy of Pediatrics (AAP) note that there are no definitive age limitations for bariatric surgery in youth, but that the majority of those who undergo surgery are adolescents [24]. There are two surgical procedures considered in adolescents (ages 13–19): Roux-en-Y gastric bypass (RYGB) and vertical sleeve gastrectomy (VSG). RYGB entails creating a small gastric pouch from the upper stomach that is connected directly to the middle portion of the small intestine, inducing both restriction and changes in absorption [19]. VSG involves removal of 80–90% of the greater curvature of the stomach, which restricts food intake [19]. VSG also leads to weight loss through delayed gastric emptying and altered neurohormonal feedback mechanisms [25,26]. VSG is now the most common bariatric procedure in both adults and adolescents [19,23,25], and VSG is currently the only type of bariatric surgery used in children younger than 13 years of age [27]. Laparoscopic adjustable gastric banding (LAGB) is performed with a device that can be easily removed, but its use is limited by the FDA to patients aged 18 years and older [19,28]. For a variety of reasons including cost, limited access to care, and patient concerns about adverse events, only 1–2 percent per year of eligible adult patients undergo bariatric surgery in the United States [22]. This percentage is even lower in the child and adolescent population [22]. Further, it is unclear if physicians refer racial/ethnic minorities, who are disproportionally affected by obesity, to multidisciplinary weight-loss centers at equal rates as their White counterparts [29]. Subsequently, if racial/ethnic minorities do ultimately attend a multidisciplinary center, it is unknown whether they pursue bariatric surgery at equal rates as their White counterparts [29].

Although bariatric surgery is a powerful tool to enable weight loss and decrease associated comorbidities (i.e., diabetes) [30], approximately 10% of patients exhibit less than expected weight loss post-surgery, despite following the prescribed diet and exercise [31]. The addition of anti-obesity medications in patients with inadequate weight loss or even weight regain after bariatric surgery appears to improve outcomes [22].

Advances in safety and efficacy of bariatric surgery in adults have led to its increasing utilization in the adolescent age group, with comparable or sometimes superior results [31]. Adolescent bariatric surgery provides long term resolution of comorbid diseases, including diabetes and hypertension, and often outperforms resolution of these measures in the adult population [30,31]. However, since adolescents are often still growing, providers should give particular attention to nutritional monitoring and to developing a transition plan to an adult bariatric care center [19,32].

The Teen-Longitudinal Assessment of Bariatric Surgery (Teen-LABS) was a large pediatric prospective, longitudinal, multi-center observational study, following 242 adolescents undergoing bariatric surgery for severe obesity. Inge et al., compared Teen-LABS data to LABS data (an adult study from which Teen-LABS study measures were derived), to compare five-year outcome data [33]. The adolescents were ≤19 years of age at the time of a bariatric procedure (67% RYGB, 28% VSG, and 6% LAGB) from March 2007 through February 2012 [34]. At baseline, 28% were early teenagers (ages 13–15), 41% were middle teenagers (ages 16–17), and 30% were late teenagers (ages 18–19) [34]. Bariatric surgery outcome differences between younger and older adolescents were few, indicating that younger adolescents should not be denied for consideration [23,35].

Remission rates of diabetes differed significantly between adults and children. Of patients with diabetes at baseline, 86% of adolescents, whereas only 53% of adults, no longer met criteria for diabetes 5 years after surgery [33]. Remission rates of hypertension also differed significantly; 68% of adolescents, but only 41% of adults, entered remission 5 years after surgery. This study suggested that adolescents had greater plasticity for reversal of obesity-related complications than adults. Diabetes remission may be modulated by greater opportunity for recovery of islet cell secretory capacity, while hypertension remission may have involved less-reversible anatomical and structural changes to the heart with age [33]. Bariatric surgery in pediatrics also improved fatty liver disease [28].

Alqahtani et al. studied the largest cohort to date of children and adolescents undergoing weight-loss surgery, with 2504 participants undergoing VSG over 10 years. Their group showed durable weight loss and maintained comorbidity resolution [27]. They were also one of the few groups to measure vertical growth over time; patients reassuringly had unaltered growth, which is of particular concern in the pediatric population [27]. However, it is important to note that long-term data was limited to approximately 25% (632 patients) of the entire cohort [36].

## 5. Architecture and Composition of Intestinal Flora in Obesity

The intestinal microbiota is a complex community of bacteria, prokaryotes, eukaryotes, and archaea [37]. There are approximately 3.8 × 10^13^ bacterial cells in and on the human body and 3.0 × 10^13^ human cells, equating the bacterial to human cell ratio to be approximately 1:1, as opposed to the frequently cited 10:1 ratio [38]. The evolution of symbiosis between the human gastrointestinal tract and its resident microbiota confers reciprocal interactions between the gut microbiome and the host, with important consequences for human health and physiology [39].

There are four predominant bacterial phyla in the human body [40]. First, the phylum Firmicutes consists of classes Bacilli and Clostridia, which are gram-positive organisms with diverse physiology (anaerobic, aerobic), and include commensal and beneficial bacteria; examples include *Lactobacillus*, *Ruminococcus*, *Clostridium*, *Staphylococcus*, *Enterococcus*, and *Faecalibacterium*. Second, the phylum Bacteroidetes consists of the class Bacteroidetes with gram-negative organisms widely distributed in the environment (i.e., soil, seawater, and the guts of animals); examples include *Bacteroides* and *Prevotella*. Third, the phylum Proteobacteria consists of classes Gammaproteobacteria and Betaproteobacteria, with gram-negative organisms that include a wide variety of potential pathogens; examples include *Escherichia* and *Pseudomonas*. Fourth, the phylum Actinobacteria consists of class Actinobacteria, with gram-positive organisms with diverse morphology; examples include *Bifidobacterium*, *Streptomyces*, and *Nocardia* [40].

The Integrative Human Microbiome Project showed dynamic changes in gut microbiota in disease states such as in pregnancy with preterm birth, inflammatory bowel disease, and diabetes [41]. Diet was also strongly associated with differences in the gut microbiota [42]. The depleted microbial biodiversity of gut microbiota in people consuming a Western diet (high in fat, sugars, and animal proteins) was associated with increasing incidence of obesity, coronary vascular disease, and metabolic syndrome [42,43]. The beneficial *Prevotella* enterotype was associated with a favorable carbohydrate-based diet (high in both complex carbohydrates and simple sugars) [44].

Adults with obesity generally had a greater Firmicutes/Bacteroidetes ratio and increased Proteobacteria compared to lean controls, although there was variation among individual studies [45]. Turnbaugh et.al. characterized the fecal microbial communities of 154 adult female monozygotic and dizygotic twin pairs (along with their mothers) for concordance with normal weight or obesity [46]. The analysis revealed a lower proportion of Bacteroidetes and a higher proportion of Actinobacteria in patients with obesity versus patients with a normal weight [46]. Children with obesity also had gut microbiota compositional and functional differences compared to lean controls, including increased proinflammatory bacterial taxa [47,48]. Similar associations were also found in mice [47,48]. Obesity was associated with a reduction in Bacteroidetes and a proportional increase in Firmicutes in mice with obesity versus lean mice, regardless of kinship [49].

Fecal samples from the same individual were much more similar to one another than samples from family members or unrelated individuals, demonstrating that temporal changes in community structure within an individual were minor compared to inter-personal differences [46]. This finding implies that future studies examining the gut microbiota, in either an observational or interventional fashion, will have the most impact if individuals are studied over time.

There are likely several mechanisms involved in the relationship between the gut microbiota and obesity, though functional studies are limited. Chierico et al. studied the composition of gut microbiota in relation to metagenome functional content in adolescents and adults with obesity compared to age-matched volunteers with normal weight [47]. The adolescents with obesity had more of an association with primary bile acid biosynthesis, steroid acid biosynthesis, fructose metabolism, mannose metabolism, galactose metabolism, butanoate metabolism, pentose phosphate metabolism, and glycolysis/gluconeogenesis. However, the adolescents with a normal weight had more of an association with secondary bile acids, steroid hormone metabolism, lipoic acid metabolism, and glycan biosynthesis and metabolism [47]. Primary bile acids are those synthesized by the liver and secondary bile acids are the result of conversion of primary bile acids by colonic bacteria [50]. Secondary bile acids influence energy expenditure and glucose homeostasis in a positive fashion via their effects on gluconeogenesis, insulin secretion, and insulin sensitivity [51]. Insulin exerts pleiotropic effects on multiple organs; it regulates body fat through a dynamic network of factors that control energy imbalance [52]. Direct measurements of gut microbiota derived metabolites also showed differences between individuals with obesity and those with a normal weight [53]. Although there was variation between studies, a meta-analysis showed that stool from adults with obesity compared to stool from adults with a normal BMI have increased levels of short chain fatty acids [54].

## 6. The Developing Microbiome Modulates Obesity Differently than in Adults

### 6.1. The Birthing Process

The first significant microbial exposure in infancy occurs at birth, microbes closely resembling microbes encountered during the birthing process colonize the infant’s gut [55,56]. Vaginally delivered infants acquire bacterial communities resembling their own mother’s vaginal microbiota, which is dominated by *Lactobacillus, Prevotella,* and *Sneathia* [57]. In contrast, Caesarian section delivered infants acquire bacterial communities resembling those of maternal skin, which is dominated by *Staphylococcus, Corynebacterium,* and *Propionibacterium* [57]. Moreover, there is differential subsequent development of the gut microbiome in infants delivered by Caesarian section compared with vaginal delivery [58]. Most notably, infants delivered by Caesarian section have overrepresentation of pathobionts, especially specific strains acquired from their mother, and underrepresentation of *Bacteroides* and *Bifidobacterium* [58]. While these differences by delivery mode are most pronounced during the first year of life, some differences persist into early childhood [59]. Furthermore, impacts to microbiome development during critical early-life periods of immune and metabolic programming have long-term health consequences [56]. This is especially important since the children of mothers who have obesity are more likely to develop obesity themselves, and babies who are born by Caesarian section are also more likely to develop obesity [60,61]. Moreover, exposure to antibiotics early in life profoundly alters the gut microbiome [62,63]. Antibiotics in early life are associated with later obesity, with animal models indicating a causal effect by the gut microbiome [56,64,65].

### 6.2. The First Three Years of Life

There is dynamic development of the gut microbiome early in life, with rapidly increasing diversity over the first few years of life [66,67,68,69]. Koenig et al. conducted an intriguing study in which one full-term male infant was chronicled with stool samples daily for 2.5 years, allowing for an in-depth look into the dynamics of a developing intestinal ecosystem in relation to known disturbances (i.e., breastfeeding, introduction of solids, antibiotic usage) [68]. The authors observed an increase in diversity over time. 16S ribosomal RNA gene sequencing of the samples showed a dramatic and sustained increase in abundance of Bacteroidetes immediately after the introduction of peas and other table foods. Bacteroidetes are specialized in the breakdown of complex plant polysaccharides [68]. Low levels of Bacteroidetes in the gut are correlated with obesity, with obesity itself potentially resulting from a diet low in plant-derived polysaccharides [42,68].

Overall, the infant gut microbiome is rapidly colonized and plays an important role in the development and education of host mammalian immune and metabolic systems. There appears to be a “critical window of opportunity” for education of these systems [70]. If that “window” is missed, and host-commensal interactions are disrupted, diseases may develop later in life, including obesity, food allergies, and asthma [56,70].

### 6.3. School-Aged Children and Adolescents Continue to Have a Dynamic Microbiome

Some studies suggest that the gut microbiome becomes relatively stable and adult-like in the first 1 to 3 years of life [66,67,68,69], but other evidence indicates that it continues to develop into the teenage years [67,71,72]. Agans et al., studied gut microbiota in adolescent children (ages 11–18) versus adults and found that the abundance of *Bifidobacterium* was significantly higher in adolescent children than in adults [72]. The authors implied that levels of *Bifidobacteria* in children decreased gradually between 2 and 18 years of age until reaching stable levels in early adulthood, rather than plummeting quickly after toddlerhood [72].

In addition, Hollister et al. studied the gut microbiome of 46 healthy pre-adolescent children (7–12 years of age) in comparison to healthy adults from the same region (Houston, TX, USA) [67]. Although healthy children and adults had similar numbers of taxa and functional genes, their compositional and functional potential differed significantly. Children had increased amounts of *Bifidobacterium*, *Faecalibacterium*, and members of Lachnospiraceae, while adults had greater amounts of *Bacteroides*. Regarding function, there were significant differences in the relative abundance of genes involved in vitamin synthesis, amino acid degradation, oxidative phosphorylation, and mucosal inflammation. Children’s gut microbiota was enriched with functions that could support ongoing development, while adult gut microbiota was predominant in functions associated with inflammation, obesity, and increased risk of adiposity.

Collectively, these results suggest that the healthy pediatric gut microbiome harbors compositional and functional aspects that differ from similarly matched healthy adults, and that the gut microbiome may undergo a more prolonged development than previously anticipated [67]. Therefore, childhood and adolescence may provide opportunities for microbiome interventions to promote health or prevent obesity, since the developing microbiome plays a more definitive role in establishing metabolic pathways involved with energy regulation [73,74,75,76,77].

### 6.4. The Developing Gut Microbiota Influences Host Metabolic Status

There are data suggesting that aberrant compositional development of gut microbiota precedes being overweight [78,79], offering new possibilities for preventive and therapeutic applications in weight management [80]. Cani et al., demonstrated that mice fed a high fat diet developed insulin resistance and inflammation by a mechanism directly dependent on lipopolysaccharide (LPS), an essential component of the cell walls of gram-negative bacteria such as *Bacteroides* [78]. They found that a high fat diet increased the proportion of LPS-containing microbiota in the gut [78]. In a subsequent study, they reported that mice fed a high fat diet had reduced amounts of *Bifidobacterium* and *Bacteroides* when compared to controls fed a standard diet [81]. Moreover, they noted increased rates of plasma LPS concentrations associated with lower *Bifidobacterium* levels in the intestinal microbiota. *Bifidobacteria* were shown to reduce intestinal endotoxin levels and upgrade mucosal barrier function [82]. The supplementation of prebiotic dietary fiber in mice fed a high fat diet increased *Bifidobacteria* in the gut, leading to a reduction of endotoxins in the blood [81]. The specialized diet was also strongly associated with improved glucose tolerance as explained by normalization of glucose-induced insulin secretion [81]. Furthermore, in later studies they also found increased gut permeability from inflammation enhanced LPS absorption [79].

The gut microbiota also influences host adiposity by energy extraction from the diet and by regulation of metabolism throughout the body [83,84]. For example, Bäckhed et al. showed that gut microbiota influenced host fat storage in mice [83]. Fasting-induced adipocyte factor (Fiaf), a circulating lipoprotein lipase inhibitor, suppression was essential for gut microbiota-induced deposition of triglycerides in adipocytes. The gut microbiota inhibited Fiaf, caused increased hepatic lipogenesis with increased lipoprotein lipase activity in adipocytes, thereby promoting storage of calories harvested from the diet into fat [83]. The above data in total suggest that restoration of the gut microbiota to a healthy state may ameliorate conditions associated with obesity and help maintain a healthy weight [85].

### 6.5. The Gut Microbiota Has a Causal Role in Obesity

Moving beyond associations, several murine models showed a causal role of the gut microbiota in obesity. Body fat, fat mass, and obesity-associated metabolic phenotypes can be transferred via gut microbiota, as shown in a study where fecal samples from human adult females with obesity were transferred into germ free mice [86]. Furthermore, early life antibiotic-induced obesity can also be transferred into germ free mice [56]. Moreover, the intestinal microbiota of mice with obesity was more effective at extracting calories from food than that of mice with a normal weight [87]. This trait that can be passed to germ-free mice via fecal microbiota transplant, thereby causing increased adiposity in those initially germ-free mice [87]. These studies did not detail any adverse side effects regarding the mice.

## 7. Prebiotics, Probiotics, Synbiotics, and Fecal Microbiota Transplant Modulate Obesity

### 7.1. Probiotics

Probiotics are live microorganisms, which when administered in adequate amounts confer a health benefit to the host, with strain-specific mechanisms of action [88]. Beneficial microbes such as *Lactobacillus, Bifidobacterium,* and *Streptococcus* are commonly used as probiotics [73]. Multiple systematic reviews and meta-analyses in humans showed that probiotics may have some benefit in combating obesity, however, data were inconsistent, with other studies showing no significant effect of probiotics in reducing BMI [89,90].

### 7.2. Prebiotics

Prebiotics are selectively fermented ingredients that confer a health benefit to the host through specific changes in the composition of or activity of the gastrointestinal microbiota [91]. Prebiotics must also meet three criteria: (1) resistance to gastric acid, bile, and digestive enzymes; (2) ability to undergo fermentation by gut microbiota; and (3) ability to stimulate the growth and/or activity of commensal gut microbiota [91]. Gut hormones such as GLP-1 play a critical role in relaying signals of nutritional and energy status from the gut to the central nervous system to control food intake [76]. GLP-1 is upregulated by prebiotics, suggesting that prebiotics may be used to control food intake [76]. Nicolucci et al. illustrated that the prebiotic of an oligofructose-enriched inulin selectively altered the intestinal microbiota, and significantly reduced body weight z-score in children with overweight and obesity compared to placebo [92]. However, Liber et al. did not show a difference in BMI when using the prebiotic of oligofructose-enriched inulin compared to placebo in children with overweight and obesity [93].

### 7.3. Synbiotics

“Synbiotics” are a combination of probiotics and prebiotics [94]. Synbiotics have the potential to induce more beneficial effects on the gut microbiota than isolated intake of pre- or probiotics. They promote the survival and implantation of live microbial dietary supplements in the gastrointestinal tract of the host [94]. A systematic review and meta-analysis by Mohammadi et al. of probiotics and synbiotics, did not illustrate any significant changes in BMI, waist circumference, body fat, fasting blood glucose, or lipid profiles before and after supplementation with probiotics/synbiotics for 4–16 weeks [95]. However, a subgroup analysis by intervention type, revealed a significant reduction in BMI with synbiotic supplementation [95].

### 7.4. Fecal Microbiota Transplant

Fecal microbiota transplant (FMT) is a process aimed to restore microbial homeostasis to altered gut microbiota by transplanting stool from a healthy donor into a recipient via pills, nasogastric/nasojejunal tubes, colonoscopy, enema, rectal tube, or sigmoidoscopy [96]. The Gut Bugs Trial was a randomized, double-blind, placebo-controlled trial assessing the efficacy of FMT to treat obesity and improve metabolism, with the primary outcome being BMI standard deviation at 6 weeks post-FMT [97]. The participants included 87 adolescents (ages 14–18) with a BMI ≥ 30 kg/m^2^. The teenagers received a single course of oral encapsulated fecal microbiota from donors of the same sex or saline placebo, with a 26-week follow-up. FMT did not show an effect on BMI, insulin sensitivity, liver function, lipid profile, inflammatory markers, blood pressure, or gut health, but it did show a reduction in abdominal adiposity. The participants faced no serious adverse events, although minor adverse events occurred, with the most common being loose stools in 10% of participants. [97]. Prior FMT studies in adults with obesity also did not show a significant change in BMI [98,99], although a temporary improvement in insulin sensitivity was demonstrated in two studies [100,101]. Conversely, two other studies reported no effects on insulin sensitivity, but showed sustained gut microbiota changes after FMT [98,99].

There are risks associated with FMT. There have been cases of infectious pathogen transmission resulting in hospitalization or even death [102,103]. Furthermore, other unintended characteristics may be transferred. For example, a woman with a normal BMI quickly gained 34 pounds after receiving a fecal transplant from her daughter with an overweight BMI for the treatment of a *Clostridioides difficile* infection [104]. However, the research team could not say with certainty that the transplant spurred the sudden weight gain [104].

## 8. Bariatric Surgery Leads to Gut Microbiota Changes in Adults

Weight loss mediated through bariatric surgery was initially thought to be a direct result of mechanical alterations causing restriction and calorie malabsorption, but the degree of enhanced metabolism after surgery cannot be explained by caloric restriction and weight loss alone [25,26,105,106]. Bariatric surgery induces changes in the anatomy of the digestive tract, gastric emptying, hormonal status, quantity and choice of ingested nutrients, and the metabolism of bile acids, which all might modify the gut microbiota composition [75,107,108]. In addition, host physiological responses include “resetting” of the body’s metabolism and hunger signals, with energy harvesting through hormonal changes [105]. Furthermore, given the established impact that the gut microbiota has on adiposity, it is likely that complex enteric microbe-host responses contribute to surgery-mediated weight loss and maintenance of weight loss post-surgery [75,105]. A systematic review by Guo et al., on human and mouse model bariatric surgery, demonstrated that microbial composition greatly changed after surgery [109]. Overall, it showed increased Bacteroidetes, Fusobacteria, Verrucomicrobia, and Proteobacteria with decreased microbial groups of Firmicutes, Clostridiales, Clostridiaceae, *Blautia*, and *Dorea* [109].

Furet et al. examined the gut microbiota of 30 adults with obesity before and after RYGB and found that gut microbiota prior to surgery showed significant depletion of *Bacteroides/Prevotella* in comparison to adults with a normal weight [80]. After RYGB there were increased amounts of *Bacteroides/Prevotella* at 3 months post-surgery, when patients exhibit most of the expected weight loss from the surgery. Those changes remained stable at 6 months post-surgery, when weight loss generally plateaus, at a level of abundance close to that of the control subjects with a normal weight. RYGB in and of itself may independently contribute to changes in gut microbiota composition by bypassing gastric acidity, causing a reduction of chloride acid influx into the intestines, and leading to increased pH. The increased pH together with downstream delivery of bile acids could contribute to the modification of the population of intestinal bacteria [80]. Bile acids exert a strong selection pressure on gut microbiota via direct and indirect antimicrobial effects [110,111]. Conversely, the microbial changes in RYGB may differ from other types of bariatric surgery procedures such as VSG or LAGB, which do not induce malabsorption with their anatomy. For example, studies following patients after RYGB reported an increase in serum bile acids, but no differences in bile acid levels are observed following VSG [112].

Davies et al. conducted a systematic review examining the clinical studies that characterized changes in gut microbiota after bariatric surgery [113]. They identified a total of 454 articles; screened 297 (title/abstract) after removing duplicates; and assessed 21 full texts for eligibility. Finally, they included 14 studies (11 prospective cohort studies, 1 randomized controlled trial, and 3 retrospective case-control studies). The studies included 222 participants who underwent bariatric surgery: 146 RYGB, 25 VSG, 14 LAGB, and 37 other kinds of bariatric surgery (30 biliointestinal bypass and 7 vertical banded gastroplasty). The majority examined gut microbiota changes after RYGB at varying durations (3 months to 12 months). In general, there was increased microbial diversity and gene richness after surgery [113]. One study identified 58 new genera in patients after RYGB, with the highest richness observed as soon as 3 months post-surgery [114]. Dramatic shifts in phylum, genera, and species composition of gut microbiota were observed after surgery [113]. There were decreases in the relative abundance of Firmicutes after VSG and increases in Proteobacteria after RYGB. Bacteroidetes were significantly increased in the gut microbiome of patients after VSG but decreased after RYGB. It is unlikely that the discordant changes in Bacteroidetes are causally linked with the metabolic changes observed after both VSG and RYGB, given similar weight loss and resolution of type 2 diabetes mellitus. In addition, common underlying species and/or strain specific changes may have occurred, which can be masked by phylum level analyses [113].

Fukuda et al. examined patients receiving VSG in terms of the gut microbiota and immune environment, including mucosal-associated invariant T cells (MAIT cells) and regulatory T cells (Treg cells) [115]. They discovered that a reduction in chronic inflammation in obesity was secondary to a change in the constituent bacterial species, diversity of gut microbiota, MAIT cells in the colonic mucosa, and effector Treg cells in the peripheral blood. An increase in diversity of gut microbiota was the postulated mechanism of action whereby VSG in those with morbid obesity led to improvements in gut microbiota balance. The increased microbial diversity then conferred an enhanced intestinal immune environment by way of increased MAIT cells in the colonic mucosa, which led to a decrease in chronic inflammation. Subsequently, an advancement in blood immune environment created a decrease in both effector Treg cells and Th1 cells [115].

There are a few limitations in these adult studies on the gut microbiota and bariatric surgery. Most were conducted using 16S ribosomal RNA gene sequencing which does not allow for strain level examination of the microbiome. Moreover, they show microbiome associations but not causal mechanisms. Further studies are needed to better delineate mechanistic pathways. Furthermore, the studies did not analyze race and ethnicity, which is important given that obesity disproportionately affects non-Hispanic Blacks and Hispanics. The disparities could be in part due to the gut microbiome, which is influenced by a multitude of factors including race and ethnicity [116]. It is also unknown whether patients who did not lose a significant amount of weight after bariatric surgery had different gut microbiota changes compared to those who did, as the current numbers were too small to make an inference [109].

Some of the most convincing evidence that the gut microbiota community can impact surgery-mediated weight loss in a causal manner comes from mice studies involving fecal transplant. Liou et al. tested a mouse model of RYGB surgery [117]. The transfer of stool from RYGB-treated mice to non-operated, germ-free mice led to weight loss in the recipient animals. In contrast, the transfer of stool from non-RYGB abdominal surgery control mice, to non-operated, germ-free mice, conferred no weight change in the recipient animals. This study was the first empirical claim that changes in gut microbiota contribute to reduced host weight after RYGB [117]. Similar findings were found in RYGB rat models [118]. However, such robust evidence for the role of the gut microbiota in the success of bariatric surgery in humans has not yet been demonstrated.

## 9. The Role of the Gut Microbiome in Pediatric Bariatric Surgery

**Figure 2:**
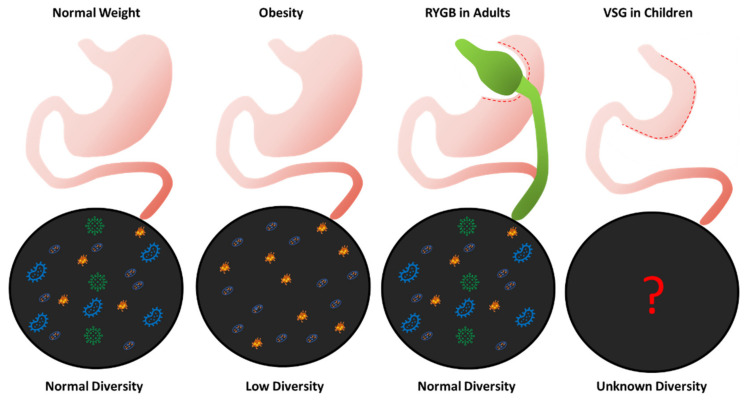
The diversity of gut microbiota in the human gut: The four images indicate the degree of gut microbial diversity in the intestines in patients with a normal weight, with obesity, and after RYGB, and the unknown nature of gut microbial diversity after VSG in children. The gut microbiota of a patient with a normal weight has a high degree of microbial diversity, which contributes to the maintenance of health. Patients with obesity have dysbiosis in their gut microbiome marked by a lack of microbial diversity. RYGB, which has been the subject of most studies examining the link between bariatric surgery and gut microbial diversity, effectively leads to weight loss and restores the dysbiosis to levels of gut microbial diversity seen in patients with a normal weight. In contrast, the most common type of bariatric surgery performed in children is VSG and the effects to the microbiome before and after VSG in children is unknown.

Gut microbiome changes after bariatric surgery in children and adolescents are largely unknown. No reported studies have involved pediatric patients (17 years of age or younger) [113]. Pediatric institutions often treat patients up to age 21, meaning that there is some age overlap with adult studies in patients aged 18 to 21. However, most of the studies focused on middle aged adults and did not provide raw data to analyze individual patients by age [113]. Furthermore, most published studies focused on patients undergoing RYGB, not VSG, the most common bariatric procedure for children and adolescents [19]. Therefore, it is important to study the gut microbiota in children and adolescents undergoing bariatric surgery, as a child’s microbiome differs from an adult, is developing, and plays a clearer role in establishing metabolic pathways that could impact energy regulation in obesity (Figure 2).

## 10. Discussion: Future Directions and Unanswered Questions

A significant knowledge gap remains regarding the specific contribution of enteric microbes to weight loss following bariatric surgery, especially in children and adolescents [105]. Additionally, it is unknown whether the pre-surgical composition of intestinal microbiota can predict whether an individual will lose a substantial amount of weight or predict who will maintain their post-surgical weight loss [105].

Understanding gut microbial changes will provide key mechanistic information regarding how weight loss in bariatric surgery is modulated. Furthermore, characterizing the gut microbiota before and after pediatric bariatric surgery may generate preliminary data for the development of microbiome-based therapeutics for the treatment of childhood obesity. These therapeutics may enhance outcomes after surgery, or even diminish the need for surgery in the future by becoming an option for weight loss themselves. 

The budding field of live biotherapeutics, which targets specific groups of microorganisms instead of replacing the entire gut microbiota landscape via FMT, could lead to more efficacious microbial therapies for obesity [119]. Companies developing live biotherapeutics currently have ongoing clinical trials for *Clostridioides difficile* infection, solid tumors, food allergies, and asthma [119,120]. Long-term, live biotherapeutics for obesity may be developed, but no clinical trials are currently underway [119,120].

## 11. Conclusions

In conclusion, early intervention is vital to curtailing the obesity epidemic, because if a child with obesity can enter adult life at a normal weight, their risk of adverse health outcomes returns to that of any other adult who possessed a normal weight their entire life. Current literature suggests a role for the gut microbiome in weight loss management. Given that children and adolescents have a dynamic gut microbiome, while an adult’s gut microbiome is more static, children and adolescents are prime targets for studies to better understand the mechanistic role of the gut microbiome in the development and treatment of obesity. A study investigating the gut microbiota of pediatric patients before and after bariatric surgery would likely help to elucidate the pathophysiology of childhood obesity, by characterizing the functional role of the gut microbiome during rapid weight loss. Such information could be used in developing microbial-based therapies to mitigate the obesity crisis.

## Data Availability

Not applicable.
